# Parkin Interacts with Apoptosis-Inducing Factor and Interferes with Its Translocation to the Nucleus in Neuronal Cells

**DOI:** 10.3390/ijms20030748

**Published:** 2019-02-11

**Authors:** Marianna Guida, Alessandra Zanon, Luigi Montibeller, Alexandros A. Lavdas, Judith Ladurner, Francesca Pischedda, Aleksandar Rakovic, Francisco S. Domingues, Giovanni Piccoli, Christine Klein, Peter P. Pramstaller, Andrew A. Hicks, Irene Pichler

**Affiliations:** 1 Institute for Biomedicine, Eurac Research, Affiliated Institute of the University of Lübeck, Via Galvani 31, 39100 Bolzano, Italy; guidamari@libero.it (M.G.); alessandra.zanon@eurac.edu (A.Z.); montibeller.luigi@gmail.com (L.M.); alexandros.lavdas@eurac.edu (A.A.L.); ladurner.judith@hotmail.com (J.L.); peter.pramstaller@eurac.edu (P.P.P.); francisco.domingues@eurac.edu (F.S.D.); andrew.hicks@eurac.edu (A.A.H.); 2 Department of Cellular, Computational and Integrative Biology and Dulbecco Telethon Institute, University of Trento, via Sommarive 9, 38123 Povo, Italy; francesca.pischedda@unitn.it (F.P.); giovanni.piccoli@unitn.it (G.P.); 3 Institute of Neurogenetics, University of Lübeck, Maria-Goeppert-Straße 1, 23562 Lübeck, Germany; aleksandar.rakovic@neuro.uni-luebeck.de (A.R.); christine.klein@neuro.uni-luebeck.de (C.K.); 4 Department of Neurology, University of Lübeck, Ratzeburger Allee 160, 23538 Lübeck, Germany

**Keywords:** Parkin, apoptosis-inducing factor, cell death, neurodegeneration, Parkinson’s disease

## Abstract

Mutations in the *PRKN* gene (encoding parkin) have been linked to the most frequent known cause of recessive Parkinson’s disease (PD), and parkin dysfunction represents a risk factor for sporadic PD. Parkin is widely neuroprotective through different cellular pathways, as it protects dopaminergic neurons from apoptosis in a series of cellular and animal models of PD. The mitochondrial protein apoptosis-inducing factor (AIF) is an important cell death effector, which, upon cellular stress in many paradigms, is redistributed from the mitochondria to the nucleus to function as a proapoptotic factor, mostly independent of caspase activity, while in normal mitochondria it functions as an antiapoptotic factor. AIF is known to participate in dopaminergic neuron loss in experimental PD models and in patients with PD. We, therefore, investigated possible crosstalk between parkin and AIF. By using immunoprecipitation and proximity ligation assays, we demonstrated a physical interaction between the two proteins. Nuclear AIF translocation was significantly reduced by parkin expression in neuroblastoma SH-SY5Y cells after exposure to an apoptogenic stimulus. These results were confirmed in primary murine cortical neurons, which showed a higher nuclear translocation of AIF in parkin-deficient neurons upon an excitotoxic stimulus. Our results indicate that the interaction of parkin with AIF interferes with the nuclear translocation of AIF, which might contribute to the neuroprotective activity of parkin.

## 1. Introduction

Loss-of-function mutations in *PRKN*, encoding the E3 ubiquitin ligase parkin, play a prominent role in familial Parkinson’s disease (PD) as they represent the most frequent known cause of recessively inherited PD [1]. In addition, parkin dysfunction represents a risk factor for sporadic PD [2]. 

Parkin is widely neuroprotective and promotes dopaminergic neuron survival in a variety of cellular and animal models [3,4,5]. Consistent with its cytoprotective role, parkin expression is transcriptionally upregulated under various stress conditions, such as endoplasmic reticulum, mitochondrial, and proteotoxic stress [6,7]. In fact, overexpression of parkin inhibits cell death in response to a variety of toxic insults, and parkin’s multivalent protective functions seem to be mostly dependent on its E3 ligase activity [8].

Parkin is normally localized to the cytosol, although a small portion of endogenous parkin was suggested to be localized within mitochondria [9], and a constitutive association of some parkin with the outer mitochondrial membrane was found [10]. Parkin is an integral regulator of mitochondrial homeostasis, controlling both degradation of dysfunctional mitochondria [11] and mitochondrial biogenesis [12]. Upon mitochondrial depolarization, it translocates to the mitochondria in a PTEN induced kinase 1 (PINK1)-dependent manner, where it mediates the ubiquitination of outer mitochondrial membrane proteins and promotes the degradation of dysfunctional mitochondria through mitophagy [11,13]. Activation and recruitment of parkin into damaged mitochondria involve PINK1-mediated phosphorylation of both parkin and ubiquitin [14,15]. Cytosolic parkin is required for the clearance of non-mitochondrial substrates like PARIS (also known as ZNF746), which acts as a transcriptional repressor of peroxisome proliferator-activated receptor gamma coactivator-1 alpha (PGC-1α), a transcriptional coactivator, and master regulator of mitochondrial biogenesis [12]. Furthermore, parkin-mediated ubiquitination leads to NF-κB activation and increased expression of the mitochondrial fusion factor OPA1, resulting in the maintenance of mitochondrial integrity, and protection from stress-induced cell death [16]. Another critical function of parkin is the inhibition of the mitochondrial translocation of the proapoptotic protein Bax to the mitochondria by its ubiquitination in the cytosol, preventing subsequent cytochrome c release [17]. In addition, several cytosolic proteins that bind parkin were identified, but no parkin-dependent ubiquitination was detected for these proteins [18,19,20]. However, additional studies are needed to further elucidate the complex pattern of parkin interactions and the resulting signaling pathways affected by such binding.

The mitochondrial-associated protein AIF (apoptosis-inducing factor) is an important cell death effector, which, like cytochrome c, is released from the mitochondria in many cellular stress paradigms. Upon release, AIF translocates to the nucleus to function as a proapoptotic factor, mostly independent of caspase activity [21,22,23]. AIF is encoded by a nuclear gene in a precursor form of 67 kDa that carries a mitochondrial localization signal in its N-terminal presequence [21]. After mitochondrial import, the presequence is cleaved off, leading to the generation of the 62 kDa mature form [24]. In this configuration, AIF is an inner mitochondrial membrane-tethered protein mostly exposed to the mitochondrial intermembrane space, although a second pool of AIF is loosely associated with the cytosolic side of the outer mitochondrial membrane [25]. The release of AIF from the mitochondria during cell death requires the cleavage of the membrane-bound protein by a cytosolic or mitochondrial protease [22,24], resulting in a truncated form of AIF (~57 kDa). AIF translocation in neuronal cells was seen after induction of various types of neuronal injury in vitro and in vivo [26,27]. Programmed cell death involving AIF, caused by a lipopolysaccharide-induced inflammatory stimulus, was shown to participate in dopaminergic neuron loss in rats [28] and other experimental PD models [29]. In addition, nuclear translocation of AIF was observed in the ventral mesencephalon of autopsy samples of patients with PD [28]. Furthermore, AIF expression was shown to be changed in peripheral blood mononuclear cells of PD patients [30], and AIF levels were higher in humans and primates as compared to mice, potentially contributing to the higher sensitivity of primates to 1-methyl-4-phenyl-1,2,3,6-tetrahydropyridine (MPTP)-induced neurodegeneration [31]. In this study, we investigated a possible crosstalk between parkin and AIF, which might contribute to the broad neuroprotective effects of parkin. Our findings suggest the inhibition of toxin-induced AIF translocation into the nucleus as a possible mechanism for the protective effect of parkin expression.

## 2. Results

### 2.1. Parkin Interacts with AIF

**Figure 1:**
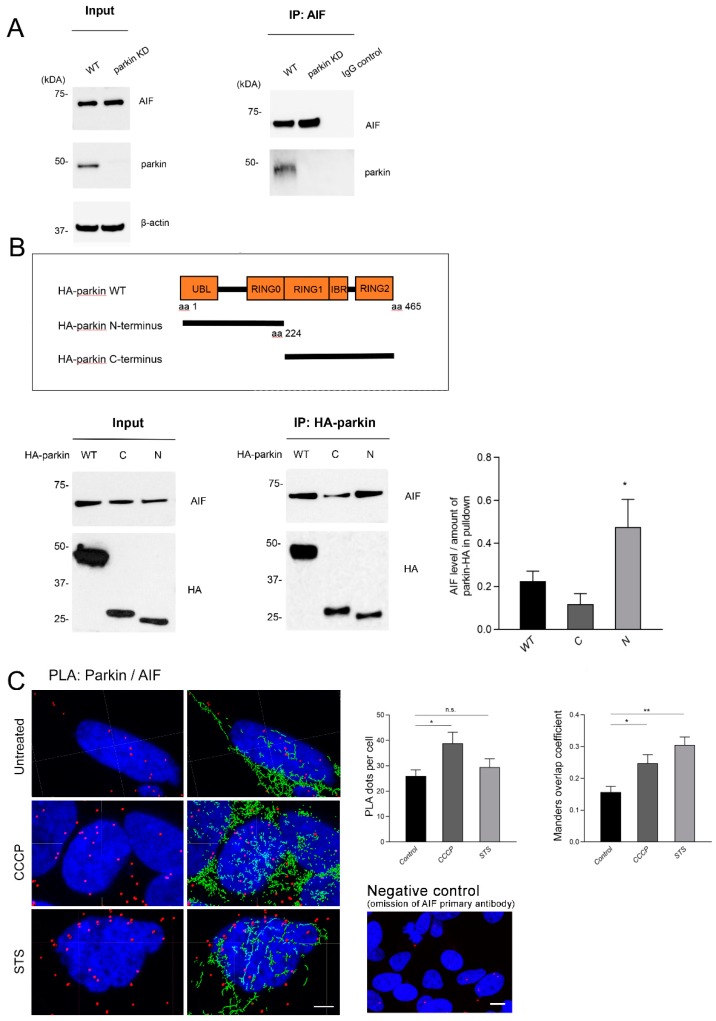
Parkin physically interacts with the apoptosis-inducing factor (AIF). (**A**) Whole-cell lysates of untreated SH-SY5Y cells with endogenous parkin levels (WT) and parkin knockdown (KD) were subjected to co-IP with an anti-AIF antibody, followed by Western blot (WB) of input and IP fractions with the indicated antibodies (the blots were probed consecutively with the antibodies). Rabbit IgG was used as negative control for the IP. Molecular mass markers are in kilodaltons (kDa). (**B**) Whole-cell lysates of HeLa cells transfected with HA-parkin (wild type, WT, C-terminal domain, C, N-terminal domain, N) were immunoprecipitated with an anti-HA antibody. WB analysis was performed with the indicated antibodies. Given the difference in expression levels of full-length HA-parkin and deletion mutants, a densitometric analysis of AIF relative to immunoprecipitated parkin is provided (mean ± SEM, *n* = 4). Two-tailed Student’s *t*-test for C- and N-terminal domains * *p* < 0.05. (**C**) SH-SY5Y cells were processed using the PLA to visualize and quantitatively assess the parkin-AIF interaction under normal culture conditions and after CCCP (10 µM, 3 h) or STS (2 µM, 3 h) treatment. The PLA signal is visualized as red dots, while DAPI-stained nuclei are shown in blue. The specificity of the PLA interaction was confirmed by performing the experiments with only one of the two primary antibodies. Exposure to CCCP increased the overall PLA signal indicating an augmented interaction between the two proteins; STS treatment did not result in a significant increase in the overall number of PLA dots per cell. A 3D reconstruction of a co-staining of the PLA signal for parkin and AIF with a mitochondrial marker (green fluorescent protein attached to a mitochondrial leading sequence, mito-GFP) was used to visualize the colocalization of the parkin–AIF complexes with the mitochondria. The colocalization was quantified by using the Manders’s overlap coefficient. Scale bar: 4 μm. * *p* < 0.05; ** *p* ≤ 0.01 compared to untreated control group (one-way ANOVA followed by Tukey’s post hoc test to correct for multiple comparisons). n.s.: not significant.

To determine whether parkin and AIF interact physically, as suggested by an interaction screen performed previously by our group [32], we employed co-immunoprecipitation (co-IP) and Proximity Ligation Assay (PLA). In whole-cell lysates extracted from SH-SY5Y cells with endogenous parkin levels cultured under basal conditions, we detected an interaction of endogenous AIF with parkin after immunoprecipitation with an anti-AIF antibody and immunoblotting with an anti-parkin antibody, which was not detected in parkin knockdown cells (Figure 1A). In reverse pulldown experiments, we immunoprecipitated overexpressed parkin tagged with a hemagglutinin (HA) epitope in whole-cell lysates extracted from HeLa cells, and detected endogenous AIF in complex with parkin (Figure 1B). Domain mapping by using N- and C-terminal deletion mutants (N: amino acids [aa] 1 to 224; C: aa 225 to 456) revealed that the interaction with AIF is mainly dependent on the N-terminal half of parkin (Figure 1B).

The interaction between parkin and AIF was further corroborated and quantitatively assessed by in situ PLA for the endogenous proteins. A co-staining of the PLA signal for parkin and AIF with a mitochondrial marker (mito-GFP) shows an increased presence of the parkin-AIF complexes (PLA dots) at the mitochondria after treatment with both the mitochondrial uncoupler carbonyl cyanide m-chlorophenylhydrazone (CCCP) and staurosporine (STS), which induces cell death. While for the CCCP treated condition, this is reflected also in a higher overall number of PLA dots per cell, in the STS treated condition, the number of PLA dots per cell does not increase compared to the untreated cells (Figure 1C).

Furthermore, the binding between parkin and AIF was detected by co-IP in mitochondrial fractions of SH-SY5Y cells with moderately overexpressed parkin, and an increased amount of co-precipitating parkin was observed upon both CCCP and STS treatment (Figure S1).

Since Parkin and AIF interact physically, we tested whether AIF is a substrate of parkin for ubiquitination. We used co-IP of whole-cell lysates of SH-SY5Y cells both overexpressing parkin and with a stable parkin knockdown, to capture endogenous AIF and to probe for ubiquitin. We used several experimental conditions, which included untreated cells and cells treated with the proteasomal inhibitor MG132 and STS separately, as well as STS and MG132 in combination. However, relative ubiquitination of AIF was not consistently altered by parkin expression levels (Figure S2).

### 2.2. Parkin Reduces AIF Translocation to the Nucleus

**Figure 2:**
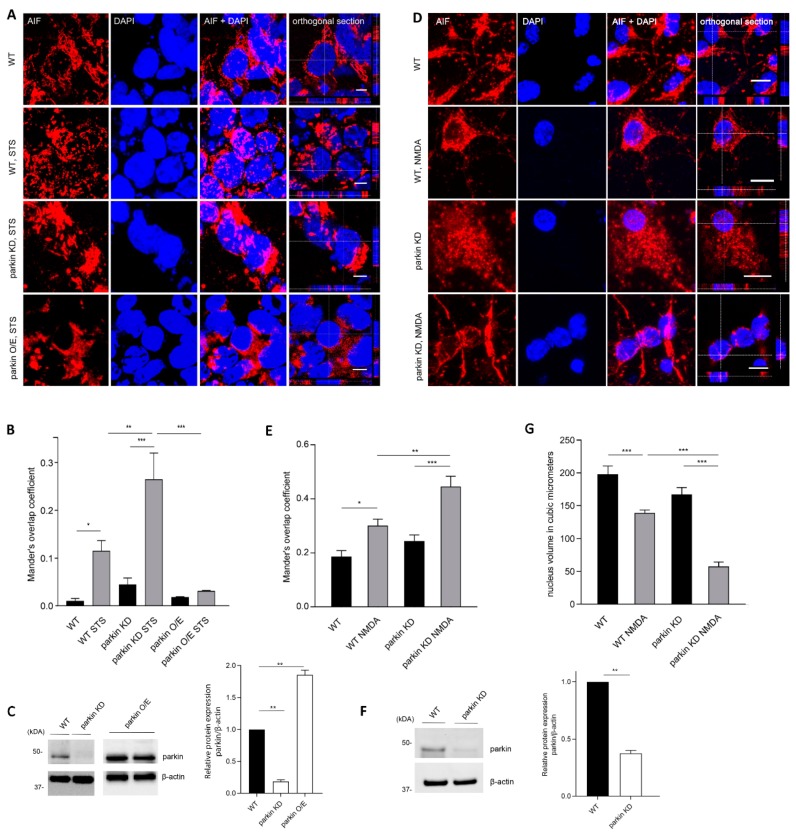
Parkin deficiency increases stress-induced translocation of AIF to the nucleus. (**A**) Representative images of AIF-nuclear colocalization in SH-SY5Y cells (WT, parkin KD, and parkin O/E) after STS treatment (3 h, 2 µM). Z stacks are provided to demarcate the nucleus. Scale bars: 5 μm. (**B**) The colocalization was quantified by using the Manders’s overlap coefficient for treated and untreated conditions. (**C**) Whole-cell lysates analyzed by WB show parkin levels for WT, parkin KD, and parkin O/E SH-SY5Y cells. Relative density values were normalized to the loading control β-actin. (**D**,**E**) Representative images of AIF nuclear translocation and its quantification in primary cortical neurons, WT (control shRNA), and parkin KD, after NMDA treatment (500 µM plus 10 µM glycin, 5 min). Scale bars: 10 μm. (**F**) Whole-cell lysates analyzed by WB show parkin levels in primary neurons, WT (control shRNA) and parkin KD. (**G**) Scoring of nuclear volume average. A lower average is indicative of more pyknotic nuclei present. Statistical differences of Mander’s overlap coefficients, nuclear volumes, and relative protein levels were calculated by one-way ANOVA followed by Tukey’s post hoc test to correct for multiple comparisons. For comparing only two groups a two-tailed Student’s *t*-test was used. Data are plotted as means ± SEM from three independent experiments (for IF, randomly chosen areas of at least two wells per condition). * *p* ≤ 0.05, ** *p* ≤ 0.01, *** *p* ≤ 0.001.

To further study the stress-protective activity of parkin, we investigated the possibility that parkin might hinder the nuclear translocation of AIF under stress conditions, and thereby attenuate its apoptogenic effect. AIF colocalized to mitochondria in untreated control cells (Figure S3). For inducing cellular stress, SH-SY5Y cells were treated with STS, a protein kinase inhibitor, which induces cell death by both caspase-dependent and independent pathways [33]. Specifically, STS has been shown to induce the mitochondrion-nuclear translocation of AIF [21,22]. To determine the translocation of AIF to the nucleus upon STS treatment in our cellular model, we examined the intracellular localization of AIF by immunofluorescence. Upon stress induction, translocation to the nucleus was detected in SH-SY5Y cells with endogenous parkin levels. Loss of parkin in cells with a stable parkin knockdown resulted in a more pronounced colocalization of AIF with the nuclei upon stimulated stress compared to cells with normal endogenous parkin levels, and cells with stable recombinant overexpression of parkin (Mander’s overlap coefficient 0.27 ± 0.05 vs. 0.11 ± 0.02 and 0.03 ± 0.001, respectively) (Figure 2A–C).

To confirm the increased AIF translocation upon parkin deficiency, we transduced primary cortical cultures with lentiviral shRNA constructs for parkin, which resulted in an efficient knockdown of parkin (60%) (Figure 2F). These cultures were exposed to N-methyl-d-aspartate (NMDA), which induces an excitotoxic stimulus to elicit delayed neuronal cell death, as assessed after incubation in neuronal complete medium for 24 h. The application of NMDA significantly increased the AIF-nuclear colocalization, and this increase was more pronounced in parkin knockdown cells as compared to wild type cells (Mander’s overlap coefficient 0.45 ± 0.038 vs. 0.30 ± 0.024 (Figure 2D,E). These data are corroborated by the quantification of nuclear volume average and a qualitative assessment of the number of pyknotic nuclei in wild type and parkin-deficient neurons, suggesting that parkin-deficient neurons exhibited increased sensitivity to NMDA treatment (Figure 2G).

## 3. Discussion

The mitochondrion-specific AIF protein is attached to the inner mitochondrial membrane, from where it projects to the mitochondrial intermembrane space [21]. A second pool of about 30% of the total protein has been identified at the outer mitochondrial membrane [34]. Within mitochondria, it is involved in cell survival, while upon toxic stimuli, a soluble form of AIF (either produced by protease-mediated cleavage or originating from the uncleaved outer mitochondrial membrane pool) translocates from the mitochondria to the nucleus, where it induces DNA fragmentation [24,34]. This caspase-independent form of cell death is named parthanatos. However, cell death can often show features linking several death types [35,36].

Parthanatos is characterized by the excessive overactivation of the nuclear enzyme Poly(ADP-ribose) polymerase-1 (PARP1), which results in the formation of high levels of poly (ADP-ribose) (PAR) polymer. PAR translocates from the nucleus to the mitochondria, where it binds AIF and facilitates its release [35,37]. While the exact mechanism of how PAR stimulates the release of AIF is not known [37], a recent study identified the macrophage migration inhibitory factor (MIF) as a cytosolic nuclease, which, by interacting with AIF, is recruited to the nucleus to facilitate DNA cleavage [38].

In the present study, we provide evidence that parkin can bind AIF and reduce its apoptogenic function by limiting its translocation to the nucleus. Our findings suggest the inhibition of AIF translocation into the nucleus as a possible mechanism for the protective effect of parkin against toxin-induced neuronal cell death. The physical interaction between the two proteins has been shown for endogenous proteins, using two different techniques under both basal culture conditions and upon induction of stress. Co-IP assays in the mitochondrial fraction and colocalization analysis of the PLA interaction signal with the mitochondria suggest a mitochondrial localization of the interaction. With parkin localized predominantly in the cytosol and AIF at the mitochondria, the interaction of parkin and AIF might be explained by the AIF pool, which is loosely associated to the cytosolic side of the outer mitochondrial membrane [34], which might allow the interaction with cytosolic proteins like parkin. Altogether, these results suggest that parkin may act directly at the mitochondrial level to reduce the AIF translocation to the nucleus upon exposure to an apoptogenic stimulus, although they do not preclude the possibility that parkin impedes this translocation in the non-mitochondrial compartment. Remarkably, endogenous levels of parkin in our cellular models seem to be high enough to contain the initialization of AIF-mediated cell death upon cellular stress induction by either STS or NMDA, as SH-SY5Y cells and mouse primary neurons with a stable downregulation of parkin are more sensitive to stress induction, showing an increased translocation of AIF to the nucleus compared to control cells. The extent of colocalization of AIF with the nucleus as quantified by using the Manders’s overlap coefficient was increased by a factor of 2.5 in SH-SY5Y cells (0.27 ± 0.05 vs. 0.11 ± 0.02) and 1.5 in cortical neurons (0.45 ± 0.038 vs. 0.30 ± 0.024) upon parkin knockdown.

Our data suggest that AIF is not ubiquitinated by parkin, and therefore our findings might be explained by the physical interaction between parkin and AIF and do not depend on parkin’s enzyme activity. On one hand, parkin is able to induce mitophagy of defective mitochondria, but is also involved in several other mechanisms to increase stress tolerance of neuronal cells. The inhibition of AIF translocation, a potent proapoptotic event, constitutes a novel mechanism participating in the stress protection of neurons by parkin. However, parkin has also been shown to sensitize toward apoptosis induced by mitochondrial depolarization but not by proapoptotic stimuli by degrading the Bcl-2 family member Mcl-1 suggesting that parkin can also show cytotoxic modes [39,40].

The results from the immunoprecipitation experiments with deletion mutants indicate that the N-terminal region of parkin includes an interaction site for AIF. The N-terminal region is composed of the Ubl domain, a linker segment and the RING0 domain, which play key roles in parkin activation. In cytosolic inactive parkin, the Ubl domain binds to RING1 in the C-terminal region blocking the E2-binding site, while the catalytic site in RING2 is blocked by RING0. Parkin activation requires large conformational changes [15,41,42]. Further investigation of the specific interaction sites between parkin and AIF in the N-terminal region and the impact of AIF binding on parkin conformation and activity are necessary, while keeping in mind that parkin likely undergoes dynamic domain movements and that the different states coexist in a dynamic equilibrium that shifts upon activation/deactivation [42]. In this respect, it is also noticeable that the densitometric analysis of AIF relative to immunoprecipitated parkin (Figure 1B) indicates that the C-terminus of parkin hinders the binding of AIF to the N-terminal region. This could be the result of an obstruction of the AIF binding site in the N-terminal Ubl or RING0 domains by intramolecular interactions to RING1 and RING2 at the C-terminus in the autoinhibited state of full-length parkin.

Overexpression/accumulation of the parkin-substrate aminoacyl-tRNA synthetase complex-interacting multifunctional protein-2, AIMP2, was reported to overactivate PARP1 leading to degeneration of dopamine neurons [43]. Inhibition of PARP1, by gene mutation or drug inhibition, suppressed dopaminergic neurodegeneration in AIMP2 overexpressing mice and in *Drosophila parkin* mutants [43,44]. Oxidative stress by increased levels of reactive oxygen species, as found in *PRKN* mutant neuronal models [45], is another stimulus able to pathologically activate PARP1 [36] and could thus lead to AIF release. Parkin-dependent ubiquitination of Bax reduces the mitochondrial accumulation of Bax under basal conditions and prevents its acute, apoptotic stress-induced (e.g., STS treatment) translocation to the mitochondria [17], where, either alone, or in combination with other proteins, it is able to induce mitochondrial outer membrane permeabilization [46]. In addition, the calcium-dependent cysteine protease calpain has been suggested to cleave AIF at the N-terminus, detaching the protein from the inner mitochondrial membrane [25]. However, the role of Bax and calpain in AIF release is debated [25,47]. Our data on the physical and functional interaction of parkin and AIF, and the existing evidence for the involvement of AIF in degeneration of dopaminergic neurons in PD, suggest that the wide neuroprotective activity of parkin can, at least in part, be attributed also to its ability to bind AIF and blunt its cell death signaling.

## 4. Materials and Methods

### 4.1. Cell Culture

Human neuroblastoma cells (SH-SY5Y, ATCC no. CRL-2266, Sesto San Giovanni, Italy) and HeLa cells (ATCC no. CCL-2) were cultured in Dulbecco’s modified Eagle’s Medium (DMEM, Sigma-Aldrich, Milan, Italy) supplemented with 10% fetal bovine serum and 1% penicillin-streptomycin (Thermo Fisher Scientific, Rodano, Italy). SH-SY5Y cells with a stable parkin knockdown or stably overexpressing parkin have been recently described [48]. SH-SY5Y cells were exposed to CCCP (10 µM, 3 h, Sigma-Aldrich) or STS (2 µM, 3 h, Sigma-Aldrich). Primary mouse neuronal cultures from the cortex were cryopreserved and cultured as described previously [49]. At days in vitro (DIV) 1, the neurons were infected with a lentiviral shRNA construct for 5’parkin (sense: GCTGTCCCAACTCCCTGATTA) and 3’parkin (sense: GATCAACATGCATCACACTCA) or a control shRNA construct with a multiplicity of infection of 2. At DIV 14, primary neurons were exposed to 500 µM NMDA (Sigma-Aldrich) plus 10 µM glycine (Sigma-Aldrich) for 5 min and then post-exposed to neuronal complete medium for 24 h before fixation and immunostaining.

### 4.2. Immunostaining and Proximity Ligation Assay

For immunocytochemical analysis, SH-SY5Y cells and primary neurons were fixed in 4% paraformaldehyde, permeabilized in 0.5% Triton X-100, and blocked in BSA. Immunostaining was performed by the addition of the primary antibody rabbit anti-AIF (ab325156, Abcam, Cambridge, UK) in blocking solution and a fluorescently labeled secondary antibody (Thermo Fisher Scientific). Nuclei were stained by the addition of 4′,6-diamidino-2-phenylindole (DAPI). Images were acquired using a Leica SP8-X confocal microscope and processed using Image J and Imaris Microscopy Image Analysis Software (Bitplane, Zurich, Switzerland).

To visualize and quantify the interaction (close proximity, <40 nm) between endogenous parkin and AIF in fixed cells, we used the PLA (“Duolink” kit, Olink Bioscience, Uppsala, Sweden) according to the manufacturer’s protocol. Mouse anti-parkin (4211, Cell Signaling, Leiden, The Netherlands) and rabbit anti-AIF (ab325156, Abcam) were used as the primary antibodies. Briefly, cells were fixed and incubated with primary antibodies and oligonucleotide-conjugated secondary antibodies, followed by the addition of the ligation solution, so that the two oligonucleotides hybridized and joined to a closed circle when they were in close proximity. Next, the amplification solution was added, initiating a rolling-circle-amplification reaction to generate a concatemeric DNA strand onto which the fluorescent detection probes subsequently hybridized, resulting in the tagging of areas where the two proteins are in close proximity. Before performing the PLA, cells were transfected with a plasmid encoding a GFP-tagged mitochondrial leading sequence (mito-GFP).

### 4.3. Co-Immunoprecipitation and Western Blot

Co-IP assays were performed in whole-cell lysates of SH-SY5Y and HeLa cells as in reference [48]. Mitochondrial fractionation was performed by using the ProteoExtract Subcellular Proteome Extraction kit (Merck, Rome, Italy). Briefly, cells or mitochondrial fractions were resuspended in a lysis buffer and incubated overnight at 4 °C with rabbit anti-AIF (ab325156, Abcam), rabbit anti-HA (sc-805, Santa Cruz Biotechnology, Heidelberg, Germany), or mouse anti-HA (H3663, Sigma-Aldrich) antibodies. Agarose beads (Roche Diagnostics, Monza, Italy) were added to the samples followed by incubation for 2 h. The proteins were released from the beads and analyzed by WB, transferred onto a nitrocellulose membrane (Biorad, Milan, Italy) and probed with the following antibodies: mouse anti-parkin (4211, Cell Signaling), mouse anti-AIF (ab110327, Abcam) or rabbit anti-AIF (ab325156, Abcam), mouse anti-β-actin (A2228, Sigma-Aldrich), mouse anti-HA (H3663, Sigma-Aldrich), mouse anti-ubiquitin (P4D1, Cell Signaling). For the ubiquitination assays, SH-SY5Y cells were lysed in denaturing lysis buffer (1% SDS) and sonicated for 10 s at 10 microns amplitude. Protein extracts were cleared by centrifugation and diluted 1:10 with non-denaturing lysis buffer. Chemiluminescence images were acquired using a Chemidoc Touch Imaging System (BioRad), and protein levels were quantified using Image Lab software (Biorad).

### 4.4. Statistical Analysis

Statistical significance was evaluated by one-way analysis of variance (ANOVA) followed by Tukey’s post hoc test to correct for multiple comparisons. A two-tailed Student’s *t*-test was used to compare data from two groups. Statistical significance was determined at *p* < 0.05.

## Supplementary Materials

Supplementary materials can be found at http://www.mdpi.com/1422-0067/20/3/748/s1.

## Abbreviations

AIF	apoptosis-inducing factor
AIMP2	aminoacyl-tRNA synthetase complex-interacting multifunctional protein-2
ANOVA	one-way analysis of variance
CCCP	carbonyl cyanide m-chlorophenylhydrazone
Co-IP	co-immunoprecipitation
DAPI	4′,6-diamidino-2-phenylindole
DMEM	Dulbecco’s modified Eagle’s Medium
DMSO	Dimethyl sulfoxide
DIV	Days in vitro
GFP	green fluorescent protein
HA	Hemagglutinin
IP	Immunoprecipitation
kDA	kilodaltons
MIF	macrophage migration inhibitory factor
MPTP	1-methyl-4-phenyl-1,2,3,6-tetrahydropyridine
NMDA	N-methyl-d-aspartate
PAR	poly ADP-ribose
PARP1	Poly(ADP-ribose) polymerase-1
PD	Parkinson’s disease
PGC-1α	Peroxisome proliferator-activated receptor gamma coactivator-1 alpha
PINK1	PTEN induced kinase 1
PLA	Proximity Ligation Assay
PRKN	Parkin
STS	Staurosporine
WB	Western blot
WT	Wild type
