# Supplementary material for: Parkin Interacts with Apoptosis-Inducing Factor and Interferes with Its Translocation to the Nucleus in Neuronal Cells
Source: Int J Mol Sci. 2019 Feb 11;20(3):748. doi: 10.3390/ijms20030748 (PMC6386878; doi:10.3390/ijms20030748)
Supplement: Supplementary file 1 [file ijms-20-00748-s001.pdf]

International Journal of
*Molecular Sciences*

MDPI

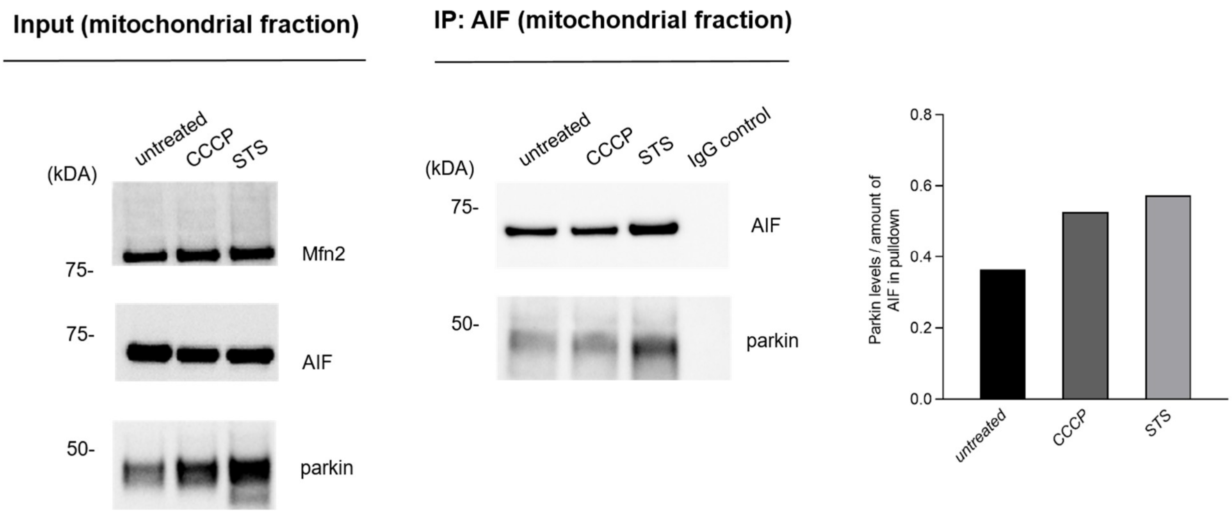


**Figure S1.** Parkin-AIF interaction in the mitochondrial fraction increases upon treatment with both CCCP and STS. Mitochondrial fractions of SH-SY5Y cells (parkin overexpression), untreated, treated with CCCP (3h, 10 μM) or with STS (3h, 2 μM) were immunoprecipitated using an antibody against AIF. IgG of the same species was used as negative control. Immunoprecipitates were analyzed by western blot with the indicated antibodies. Molecular mass markers are in kilodaltons (kDa). A densitometric analysis of AIF relative to immunoprecipitated parkin is provided.

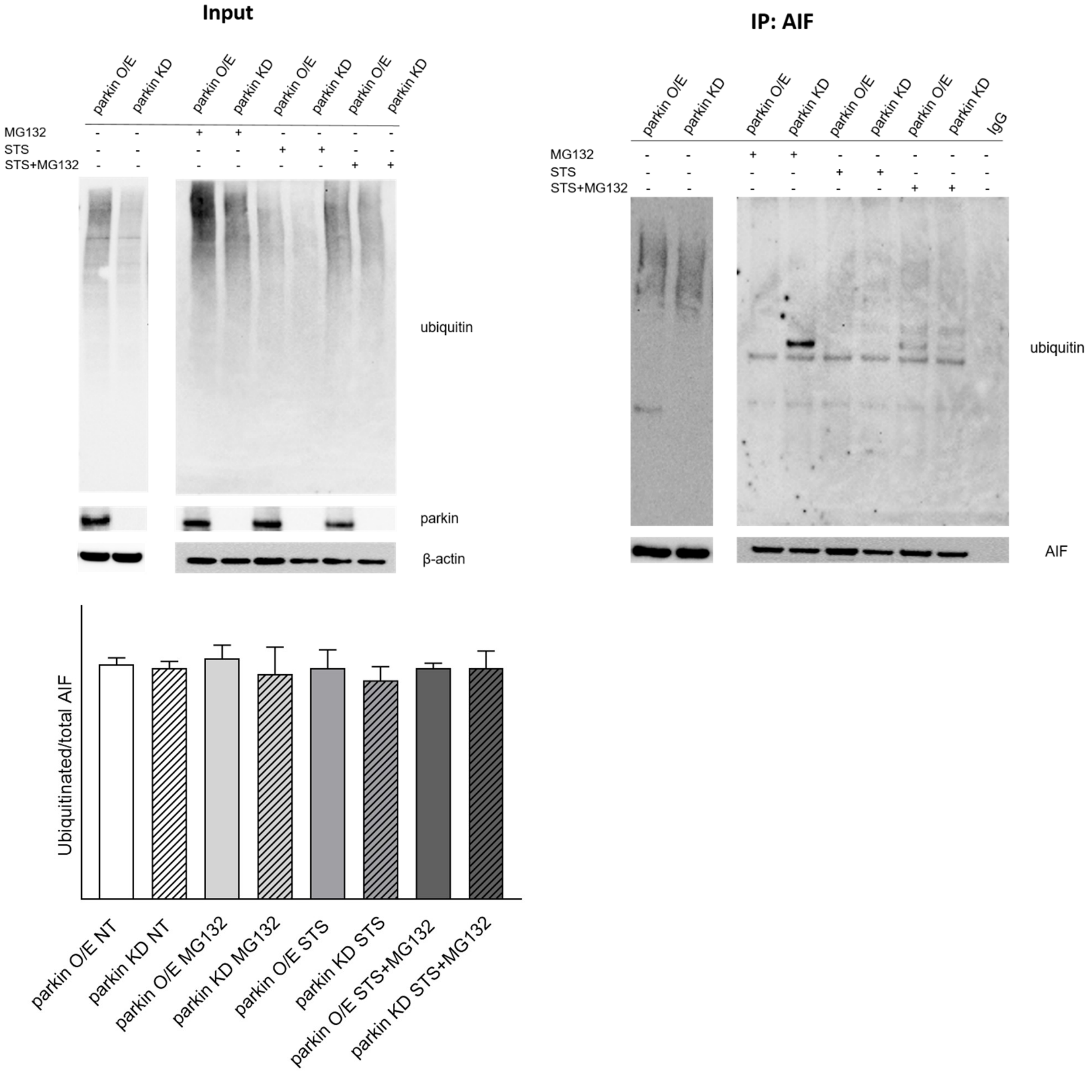


**Figure S2.** Whole cell lysates of SH-SY5Y cells (parkin overexpression, O/E; parkin knockdown, KD), untreated, treated with MG132 (6h, 10 μM), with STS (3h, 2 μM) or with STS+MG132) were immunoprecipitated under denaturing conditions using an antibody against AIF. IgG of the same species was used as negative control. Immunoprecipitates were analyzed by western blot using an antibody against ubiquitin. Densitometric analyses of ubiquitinated AIF relative to the amount of AIF in the pulldown (mean ± SEM, n=3).

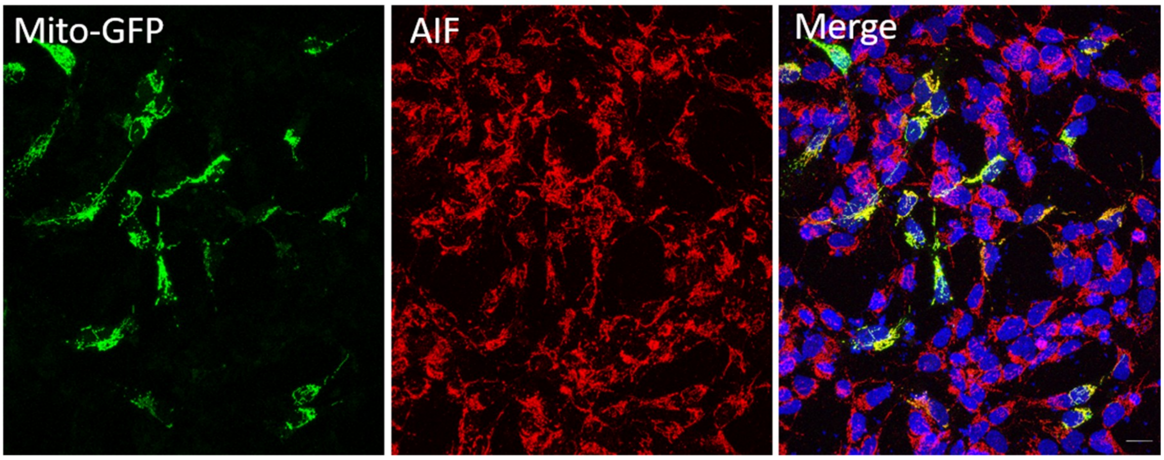


**Figure S3.** Representative image of AIF-mitochondria colocalisation in SH-SY5Y cells in the untreated conditon. Cells were transfected with green fluorescent protein attached to a mitochondrial leading sequence (mito-GFP). Immunostaining was carried out with an anti-AIF antibody (red).
